# Biophysical Characterisation of Neuroglobin of the Icefish, a Natural Knockout for Hemoglobin and Myoglobin. Comparison with Human Neuroglobin

**DOI:** 10.1371/journal.pone.0044508

**Published:** 2012-12-03

**Authors:** Daniela Giordano, Ignacio Boron, Stefania Abbruzzetti, Wendy Van Leuven, Francesco P. Nicoletti, Flavio Forti, Stefano Bruno, C-H. Christina Cheng, Luc Moens, Guido di Prisco, Alejandro D. Nadra, Darío Estrin, Giulietta Smulevich, Sylvia Dewilde, Cristiano Viappiani, Cinzia Verde

**Affiliations:** 1 Institute of Protein Biochemistry, CNR, Naples, Italy; 2 Departamento de Química Biológica, Facultad de Ciencias Exactas y Naturales, Universidad de Buenos Aires, Ciudad de Buenos Aires, Argentina; 3 Departamento de Química Inorgánica, Analítica y Química Física/INQUIMAE-CONICET, Facultad de Ciencias Exactas y Naturales, Universidad de Buenos Aires, Ciudad Universitaria, Pabellón 2, Buenos Aires, Argentina; 4 Department of Physics, University of Parma, NEST Istituto Nanoscienze-CNR, Parma, Italy; 5 Department of Biomedical Sciences, PPES, University of Antwerp, Universiteitsplein 1, Wilrijk, Belgium; 6 Dipartimento di Chimica “Ugo Schiff”, Università di Firenze, Sesto Fiorentino (FI), Italy; 7 Facultat de Farmacia, Departament de Fisicoquímica and Institut de Biomedicina, Universitat de Barcelona, Barcelona, Spain; 8 Department of Biochemistry and Molecular Biology, University of Parma, Parma, Italy; 9 Department of Animal Biology, University of Illinois, Urbana, Illinois, United States of America; 10 Departamento de Fisiología, Biología Molecular y Celular, Facultad de Ciencias Exactas y Naturales, Universidad de Buenos Aires, Ciudad de Buenos Aires, Argentina; 11 Consorzio Interuniversitario di Ricerca in Chimica dei Metalli nei Sistemi Biologici, Bari, Italy; University of Leeds, United Kingdom

## Abstract

The Antarctic icefish *Chaenocephalus aceratus* lacks the globins common to most vertebrates, hemoglobin and myoglobin, but has retained neuroglobin in the brain. This conserved globin has been cloned, over-expressed and purified. To highlight similarities and differences, the structural features of the neuroglobin of this colourless-blooded fish were compared with those of the well characterised human neuroglobin as well as with the neuroglobin from the retina of the red blooded, hemoglobin and myoglobin-containing, closely related Antarctic notothenioid *Dissostichus mawsoni*. A detailed structural and functional analysis of the two Antarctic fish neuroglobins was carried out by UV-visible and Resonance Raman spectroscopies, molecular dynamics simulations and laser-flash photolysis. Similar to the human protein, Antarctic fish neuroglobins can reversibly bind oxygen and CO in the Fe^2+^ form, and show six-coordination by distal His in the absence of exogenous ligands. A very large and structured internal cavity, with discrete docking sites, was identified in the modelled three-dimensional structures of the Antarctic neuroglobins. Estimate of the free-energy barriers from laser-flash photolysis and Implicit Ligand Sampling showed that the cavities are accessible from the solvent in both proteins.

Comparison of structural and functional properties suggests that the two Antarctic fish neuroglobins most likely preserved and possibly improved the function recently proposed for human neuroglobin in ligand multichemistry. Despite subtle differences, the adaptation of Antarctic fish neuroglobins does not seem to parallel the dramatic adaptation of the oxygen carrying globins, hemoglobin and myoglobin, in the same organisms.

## Introduction

Vertebrate neuroglobin (Ngb) is present in neuronal cells and its expression results in neuroprotection against the deleterious effects of hypoxia and ischemia [1], [2], even though these observations have been recently debated [3]. The available data also suggest that Ngb may play an important role in neuronal protection against reactive oxygen and nitrogen species [4]–[7]. Indeed, previous studies have demonstrated that Ngb can function as a nitrite reductase to form nitrogen monoxide (NO) [8] by a reaction similar to that of myoglobin (Mb) [9]–[10]. Furthermore, Jayaraman et al (2011) recently showed that hypoxia stress induces post-translational modifications, e.g. phosphorylation of Ngb, increasing its nitrite reductase activity [11].

Antarctic icefish of the family Channichthyidae lack the genes encoding hemoglobin (Hb) and, in many species, Mb. The blood of *Chaenocephalus aceratus* is colourless and nearly transparent, and iron poor [12]. Oxygen is carried in physical solution in the plasma, providing ∼10% of the carrying capacity of red-blooded Antarctic fish species. The lack of Hb is accompanied by dramatic cardiovascular modifications compared to similar-sized red-blooded notothenioids [13]–[15]. *C. aceratus* also fails to produce cardiac Mb, and the mitochondrial density of cardiomyocytes is greatly increased as compared to red-blooded, Mb-expressing fish [16]. The expansion of cellular mitochondrial density in *C. aceratus* may enhance oxygen flux in the heart [17], for compensating the absence of Mb [18], [19]. Moreover, recent studies highlight how the loss of Hb and Mb, their associated NO-oxygenase activity and the subsequent increase of NO circulating levels with respect to the other Antarctic red-blooded fishes could explain the unique cardiovascular and physiological traits evolved in icefish [15], [20]. Therefore, the icefish may be a valuable system for understanding the homeostatic and signal transduction pathways involved in the response to the lack of respiratory hemoproteins.

The discovery of the Ngb gene in the icefish [21], [22] suggests that, although Hb and Mb are missing, the protein may have important implications in the physiology of the brain of these organisms. In order to investigate the influence of the lack of Hb and Mb on the function of Ngb, we have cloned, over-expressed and purified Ngb from the brain of *C. aceratus* and, in parallel, from the retina of the closely related Antarctic red-blooded fish *Dissostichus mawsoni* (belonging to the same suborder Notothenioidei), which shares 98% amino-acid sequence identity with icefish Ngb [23]. This study has also called for a detailed comparison with the well characterised human protein, which shares 54% amino-acid sequence identity with Antarctic fish Ngbs [23].

Although the R and T canonical structures of Antarctic fish Hbs have been shown to be very similar to those of human Hb (HbA), Antarctic fish Hbs display different functional properties compared to HbA, e.g. very low-oxygen affinity [24] and high auto-oxidation rate [25], [26]. Given these differences, we thoroughly characterised Antarctic fish Ngbs to highlight possible divergences in the functional/structural properties with respect to the human protein. In contrast to expectation, the structural/functional properties of Ngb are maintained in the two fish and strongly resemble those of human Ngb, suggesting an essential, conserved role. However, human Ngb is an intracellular protein and does not cross cell membranes, whereas zebrafish Ngb seems endowed with cell-penetrating capability [27].

Resonance Raman (RR) spectroscopy, auto-oxidation kinetics, Molecular Dynamics Simulations (MDS), and Laser-Flash Photolysis experiments were carried out in a combined fashion, to gain insight into these proteins.

Differences in the reactivity with exogenous ligands and the ability to retain them for a longer time in multiple cavities with alternative exchange pathways between the solvent and the protein matrix are significant in comparison with human Ngb. These results appear relevant in the biological context of cold-adapted fish that lack oxygen-transport proteins, and are totally or partially devoid of Mb.

## Materials and Methods

### Site-directed mutagenesis

Cloning and sequencing of Ngb cDNA are reported in the Supporting Information (Text S1). Three mutations were made for crystallisation purposes on cDNA of Ngb resulting in the replacement of Cys51(CD5), Cys57(D6), and Cys121(G15) with Ser, using the QuikChange™ site-directed mutagenesis method (Stratagene). The Ngb mutants *C. aceratus* and *D. mawsoni* bearing the Cys→Ser substitutions were named *C. ace*Ngb* and *D. maw*Ngb*, respectively. These mutants were used in the spectroscopic characterisation due to their stability and for comparison with X-ray structure data. Control experiments (data not shown) indicated no significant difference with the wild-type.

### Expression and purification of Ngb

The recombinant expression plasmid was successfully transformed in the *Escherichia coli* strain BL21(DE3)pLysS (Invitrogen). Growth of the transformed bacterium and over-expression of mutants *C. ace*Ngb* and *D. maw*Ngb* were performed as described for wild-type (wt) human Ngb [28]. After expression, the cells were harvested and resuspended in lysis buffer [50 mM Tris-HCl pH 8.0, 2 mM EDTA, 1 mM phenylmethylsulfonyl fluoride (PMSF), 0.5 mM dithiothreitol (DTT)]. The resuspended cells were exposed to three freeze-thaw steps and sonically disrupted. The extract was clarified by centrifugation at low (10 min at 10,700× g, 4°C) and high (60 min at 105,000× g, 4°C) speed centrifugation and fractionated with ammonium sulfate. The 40–60%-ammonium-sulfate pellet was dissolved in 5 mM sodium phosphate pH 8.5 and dialysed. A DEAE-Sepharose Fast-Flow column (Amersham Biosciences) was equilibrated in the same buffer and bound Ngb was eluted with 5 mM sodium phosphate pH 8.5, 300 mM NaCl. The dialysed and concentrated material was loaded on a Hitrap™ DEAE fast-flow column (GE Healthcare) and the protein was eluted using a gradient (buffer A: 5 mM sodium phosphate pH 8.5; buffer B: 5 mM sodium phosphate pH 8.5, 1 M NaCl; 25 min 100% A, linear gradient in 40 min to 60% B). Eluted Ngb was dialysed against gel-filtration buffer, 50 mM Tris-HCl pH 8.5, 150 mM NaCl, 0.5 mM EDTA and concentrated using a Stirred Cell (Cat nr 5122, Millipore) under 2-bar air pressure. The concentrated material was run on a Superdex ™75 column (1.5×100 cm) in gel-filtration buffer.

### Electronic absorption spectroscopy

Electronic absorption spectra were measured with a double-beam Cary 5 spectrophotometer (Varian, Palo Alto, CA, USA) using a 5-mm NMR tube or a 1-cm cuvette, and a 600-nm/min scan rate. Spectra were recorded both before and after RR measurements. No degradation was observed under the experimental conditions employed. Protein samples (30–35 µM) were prepared in 20 mM Tris-HCl pH 7.6.

### Autoxidation

In order to assess and compare the stability of the oxygenated forms of *C. ace*Ngb*, *D. maw*Ngb* and human Ngb, their autoxidation rate was measured at 20°C. The proteins were previously reduced using the Hayashi reducing system [29] in a helium atmosphere in 100 mM phosphate pH 7.0. Once reduction was complete, the low-molecular-weight components of the Hayashi system were quickly removed by subsequent cycles of concentration and dilution using Vivaspin filtration devices (Sartorius Stedim Biotech GmbH, Goettingen, Germany). This step was performed at low temperature, which dramatically slows down heme autoxidation. The protein was then warmed to 20°C, and oxidation was followed through time-resolved spectra using a Cary 400 spectrophotometer (Varian, Inc).

### Resonance Raman (RR) spectroscopy

The RR spectra were obtained by excitation with the 413.1-nm line of a Kr^+^ laser (Coherent, Innova 300 C, Santa Clara, CA). Backscattered light from a slowly rotating 5-mm NMR tube was collected and focussed into a triple spectrometer as previously reported [30]. To improve the signal/noise ratio, a number of spectra were accumulated and summed only if no spectral differences were noted. The RR spectra were calibrated with indene, carbon tetrachloride, dimethyl sulfoxide and pyridine as standards to an accuracy of ±1 cm^−1^ for intense isolated bands.

The ferrous form and its CO- and oxy-adducts were prepared as described previously [26], [31]. The oxy-samples were cooled by an external flow of cold nitrogen, the laser beam was focussed on the sample using a cylindrical lens to minimise photolysis, and 3.2 mM DTT was added in order to avoid fast oxidation of the oxy form.

### CO-dissociation kinetics

Kinetics of CO replacement by NO to determine the *k_OFF_* of CO were measured on the CO complexes of human Ngb, *C. ace*Ngb* and *D. maw*Ngb* using a thermostatted stopped-flow apparatus (Applied Photophysics, Salisbury, UK). Solutions containing 10 µM Ngb in a 100 mM sodium phosphate, 1 mM DTT at pH 7.0 were degassed in a helium atmosphere, reduced with an equimolar concentration of sodium dithionite and briefly exposed to pure CO. Excess CO was finally removed by flushing with helium for 30 minutes. The entire process was followed by UV-visible absorption spectroscopy using gas-tight cuvettes endowed with a reservoir for gas equilibration [32]. A NO solution was prepared by anaerobically dissolving the NO donor MAHMA NONOate (Sigma Aldrich) in a solution containing 100 mM sodium phosphate at pH 7.0. The exact concentration of NO was then determined as 200 µM by titration with human deoxy-Hb. Displacement of CO by NO was monitored at 415 nm and 20°C.

### Classical Molecular Dynamics Simulations (MDS)

MDS were performed as described before [23], [33]. Briefly, the model of wt *C. aceratus* Ngb was generated with the Modeller9 program [34], using the human X-ray structure (PDB entry 1OJ6,16) as a template. For five- and six-coordinated states, protonation of distal HisE7 and proximal HisF8 was chosen to be in the N_δ_ position. For ligand-bound states (either oxygen- or CO), distal HisE7 was protonated in the N_ε_ atom. All simulations were performed at 300 K and 1-bar pressure using Berendsen thermostat and barostat. The Amber99 force field (ff99SB) was used for all residues, whereas parameters previously developed and thoroughly tested [35], [36] were used for the heme. All simulations were performed with the PMEMD module of the AMBER9 package [37]. After equilibrating the MD run of the six-coordinated *D. mawsoni* Ngb for 20 ns, the other structures were generated by deleting the His-Fe bond (five-coordinated state) and/or introducing the two point mutations that differentiate the two Antarctic fish Ngbs [23], followed by several ns equilibration runs. Ligand bound Ngb structures were generated by introducing CO atoms to the five-coordinated state followed by several ns equilibration runs. For each structure, 40-ns MD production runs were analysed. Frames were collected at 1-ps intervals, which were subsequently used to analyse the trajectories.

### Implicit Ligand Sampling (ILS)

The ILS method computes a regularly spaced grid (with a spacing of 0.5 Å) placing a ligand in each grid point to calculate the free energy associated with the probability of having the ligand at that position. The MDS were run in the absence of explicit ligands, assuming that diatomic ligands interact weakly with the protein. The parameter set for CO (εO: −0.12 kcal mol^−1^, εC: −0.11 kcal mol^−1^, R_min,O_/2: 1.70, R_min,C_/2: 2.10, *l*_bond_:1.13) was taken from [38]. The ILS free energy was computed using a total of 5000 frames from a 40-ns MDS and 20 rotamers per grid point. MD runs were described in [23] for fish Ngb and in [33], [39] for human Ngb.

### Laser-flash photolysis

Human wt Ngb, *C. ace*Ngb*, and *D. maw*Ngb* were diluted in 100 mM phosphate pH 7.0 to a final concentration of 40 µM and incubated overnight with 10 mM DTT. The samples were then reduced under anaerobic conditions with sodium dithionite at a final concentration of 10 mM and finally equilibrated at either 1 atm or 0.1 atm CO in a gas-tight cuvette. The laser-flash-photolysis setup has been described elsewhere [40]. Photolysis was achieved with the second harmonic of a Q-switched nanosecond Nd:YAG laser (Spectron Laser). Absorbance changes were monitored using a monochromatic cw output of a 150 W Xe arc lamp coupled to a 0.25-m monochromator (AMKO gmbh). The transient absorbance traces were measured through a second 0.125-m monochromator (77250, LOT-Oriel) with a 5-stages photomultiplier (Applied Photophysics). The voltage signal was digitalised by a digital oscilloscope (LeCroy Waverunner 104-Xi, 5 GS/s; 1 GHz). A custom dichroic filter (Omega optical) was positioned between the exit slit of the monochromator and the photomultiplier to remove residual stray light from the pump laser. A fast shutter (Vincent Associates, Uniblitz VS35 controlled by the driver VMM-T1) was positioned between the output of the first monochromator and the sample holder. The synchronisation of the laser and the shutter was controlled by a digital delay generator (Berkeley Nucleonics). The sample holder was accurately temperature controlled with a Peltier element, allowing temperature stability of at least 0.1°C (Flash100, Quantum Northwest).

## Results and Discussion

### Electronic absorption spectroscopy

**Figure 1:**
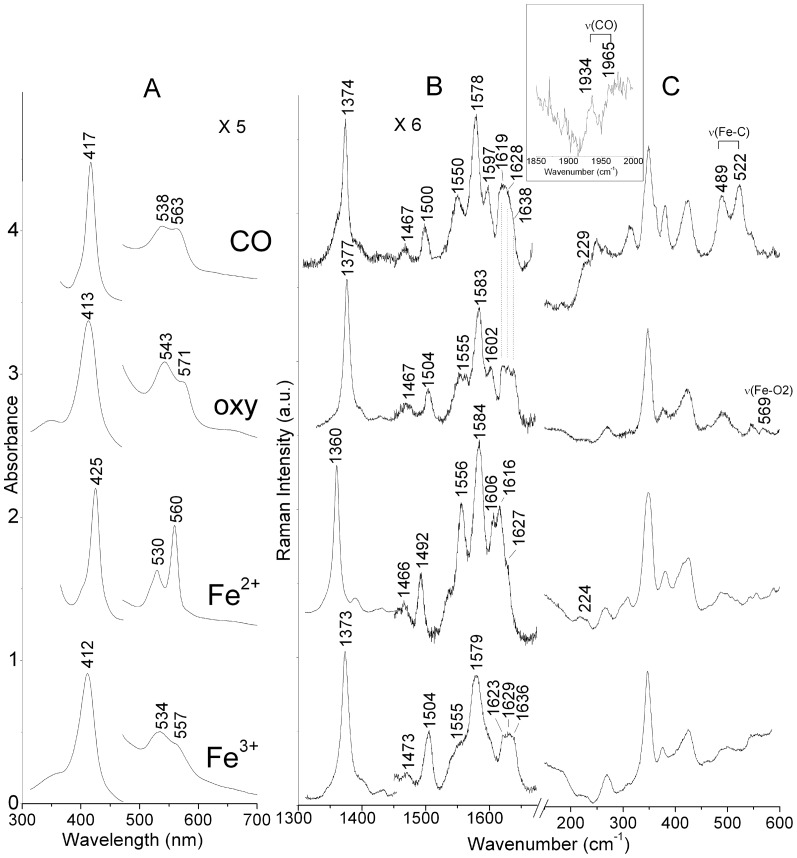
UV-visible and RR spectra of *C. ace*Ngb*. UV-visible (A) and RR spectra of Fe^3+^, Fe^2+^, oxy, and CO complex of *C. ace*Ngb* in the high (B) and low (C) frequency regions. Experimental conditions: **A.** Scan rate of 600 nm/min. **B.** 413.1 nm excitation wavelength, 1.2 cm^−1^ resolution. Fe^3+^: 10 mW laser power at the sample, average of 20 spectra with 240-sec integration time. Fe^2+^: 10 mW laser power at the sample, average of 10 spectra with 78-sec integration time. Oxy: 1 mW laser power at the sample, average of 30 spectra with 180-sec integration time. CO: 900 µW laser power at the sample, average of 7 spectra with 160-sec integration time. The intensities are normalised to that of the ν_4_ band. Spectra have been shifted along the ordinate axis to allow better visualisation. **C.** Experimental conditions in panel B. Fe^3+^: average of 12 spectra with 240-sec integration time. Fe^2+^: average of 8 spectra with 300-sec integration time. Oxy: average of 10 spectra with 80-sec integration time. CO: average of 14 spectra with 522-sec integration time. Spectra have been shifted along the ordinate axis to allow better visualisation. The intensities are normalised to that of ν_7_ (not shown). In the inset: RR spectra of the CO adducts in the ν(CO) stretching region, average of 3 spectra with 3600-sec integration time, and 3.3 cm^−1^ spectral resolution.

The UV-visible absorption spectra of ferric and ferrous *C. ace*Ngb* are typical of six-coordinated low-spin hemes (6cLS) (Figure 1, panels A and B). In particular, the UV-visible spectra of the ferric (Soret band at 411 nm, β and α bands at 534 and 557 nm, respectively) and deoxy ferrous (Soret band at 425 nm, β and α bands at 530 and 560 nm, respectively) forms unequivocally indicated the presence of a bis-His heme-iron coordination, in analogy with other Ngbs [41]. The spectra of *D. maw*Ngb* and *C. ace*Ngb* were almost identical (1 nm shift of the Soret band in the ferric form); the spectroscopic data of *D. maw*Ngb* are reported in the Supporting Information (Figure S1 and Figure S2).

Upon addition of oxygen and CO to the ferrous form, the diatomic ligands replaced distal His and gave rise to the oxy (Soret at 413 nm, β and α at 543 and 571 nm, respectively) and CO (Soret at 417 nm, β and α at 538 and 563 nm) adducts [41]–[43]. The oxygenated proteins remained stable over the time scale necessary to acquire absorption and RR spectra.

### Autoxidation

Autoxidation of the proteins (Figure S3) was observed to occur with a single exponential relaxation with time constants τ = 39±1 min (*C. ace*Ngb*) and τ = 68±3 min (*D. maw*Ngb*) at 20°C. By comparison, autoxidation of human oxy Ngb occurred at the same temperature with a time constant of 49±1 min. At 4°C the reaction was slowed down to a τ = 909 min for *C. ace*Ngb*, as reported in the Supporting Information.

In spite of the stabilisation of the oxygenated complex by distal His, the observed autoxidation rates in *C. ace*Ngb* and *D. maw*Ngb* are higher than those typical for oxygen-transport proteins, e.g. tetrameric human HbA (time constant ≈8×10^3^ min) and horse-heart Mb (time constant ≈12×10^3^ min) [44]. This further supports a role other than simply oxygen transport or storage, similar to human Ngb [41]. It has been suggested that inactivation of the protein by relatively high autoxidation rate would be overcome by a specific Ngb reductase, similar to the met-Mb reductase described in heart tissue [29]. While such reducing enzyme may also be available in these fish, efforts to identify and isolate it have been unsuccessful so far.

### Resonance Raman spectroscopy

The RR spectra of *D. maw*Ngb* and *C. ace*Ngb* were almost identical and the complete assignment of RR core size bands is reported in the Supporting Information (Table S1). In the spectrum of the oxy *C. ace*Ngb*, the band at 568 cm^−1^ (Figure 1, panel C), absent in the RR spectra of the ferrous deoxy form and of the Fe^2+^-CO adduct, was assigned to the ν_(Fe-O2)_ stretching mode. Its frequency, similar to that of mouse Ngb (571 cm^−1^) [42], suggested the presence of H-bond interaction between oxygen and distal His. Further insight into the distal cavity was gained by the study of CO bound *C. ace*Ngb* (Fe^2+^). In fact, heme-bound CO is a sensitive probe for investigating distal effects on ligand binding by heme proteins, since back-donation from the Fe d_π_ orbital to the CO π* orbitals is modulated by polar and H-bond interactions with protein residues [45], [46]. As back-donation increased, the Fe-C bond strengthened whereas the CO bond weakened, thereby increasing the ν_(Fe-C)_ vibrational frequency and decreasing the ν_(CO)_ frequency. In analogy with the Fe^2+^-CO complexes of human and mouse Ngbs [42], [43], the isotopic shift observed in the ^13^CO adduct (Table 1) allowed to identify two conformations of the Fe^2+^-CO unit in the *C. ace*Ngb* Fe^2+^-CO adduct (Figure 1, panel C). One arose from an ‘open’ conformation (Form 1) of distal His, preventing the H-bond with CO [ν_(Fe-C)_ and ν_(CO)_ at 489 and 1965 cm^−1^, respectively], and the other corresponded to a ‘closed’ conformation (Form 2) where the close proximity of dissociated distal His to CO strongly stabilised the complex, as suggested by ν_(Fe-C)_ and ν_(CO)_ at 522 and 1934 cm^−1^, respectively. Thus, distal His can adopt two conformations, in agreement with previous findings in human wt Ngb and its H64V mutant [43]. Similar open and close conformers were detected also by FTIR on the CO complex of wt Ngb [47]. Moreover, a third conformer (Form 3), weakly H-bonded with a distal residue [ν(Fe-C) and ν(CO) at 505 e 1956 cm^−1^, respectively], has recently been identified in the RR spectra of the CO adduct of recombinant human Ngb [48]. The comparison of the ν_(Fe-C)_ and ν_(CO)_ frequencies in different Ngbs-CO adducts reported in Table 1 clearly indicated that the exogenous ligand binds the proteins in a similar manner.

**Table 1:** ν(Fe-C) and ν(CO) frequencies (cm^−1^) of the Fe^2+^-CO adduct of several Ngbs.

	Form 1 (no H-bond)		Form 3 (weak H-bond)		Form 2 (strong H-bond)		*Reference*
	ν_(Fe-C)_	ν_(CO)_	ν_(Fe-C)_	ν_(CO)_	ν_(Fe-C)_	ν_(CO)_	
C. aceNgb*	489 (485)	1965 (1918)			522 (518)	1934 (1888)	This work
D. mawNgb*	489 (485)	1965 (1918)			522 (518)	1934 (1888)	This work
*Mouse*	492	1969			523	1933	[42]
*Human*	494	1972	505	1956	521	1932	[43], [46]

The frequencies obtained for the ^13^CO-adducts are given in parentheses.

Information on the proximal heme cavity can be obtained by the frequency of ν_(Fe-His)_ stretching mode being very sensitive to the interaction between the proximal ligand and the distal cavity residues [49]. However, the band was only present in the RR spectra of ferrous five-coordinated hemoproteins, giving rise to a strong band at 200–250 cm^−1^, but it is absent in the spectra of six-coordinated forms of ferrous hemoproteins [50]. Therefore, the ν_(Fe-His)_ stretching mode could not be detected in the RR spectra of ferrous *C. ace*Ngb*; the band at 224 cm^−1^ (Figure 1, panel C) also observed in human wt Ngb [43], [48] was assigned to a γ_24_ in analogy to cytochrome *c* [49]. Unlike the previous experiment, where upon CO photolysis the formation of the five-coordinated high-spin (5cHS) forms was clearly shown by the appearance of the RR ν_3_ mode at 1470 cm^−1^ [42], photolysis of the *C. ace*Ngb*-CO complex (using 25 mW, exc. 413.1 nm) was followed by immediate distal-His rebinding to the heme iron, giving rise to a bis-His 6c-LS heme (ν_3_ = 1492 cm^−1^, Figure S4). As a consequence, in the low-frequency region, while the ν_(Fe-C)_ stretching mode at 522 cm^−1^ decreased in intensity, no new band due to the ν_(Fe-His)_ stretch was observed. In a similar fashion, no ν_(Fe-His)_ stretch was observed in photolysed human Ngb-CO (data not shown); however, its ν_(Fe-His)_ has been recently assigned by time-resolved [48] and steady-state RR experiments on the distal variant H64V [43] at 221 cm^−1^, a frequency similar to that observed in horse-heart Mb [51].

Our results reveal that, similar to other Ngbs, strong polar interactions with distal pocket residues stabilise the bound ligand, as shown by RR spectra of CO and oxygen complexes.

### CO-dissociation kinetics

CO-dissociation kinetics in *D. maw*Ngb* and human Ngb (Figure S5) are homogeneous, with estimated *k_OFF_* of 0.164±0.001 s^−1^ and 0.173±0.001 s^−1^, respectively. CO dissociation from *C. ace*Ngb* appears more heterogeneous, with the main component corresponding to a *k_OFF_* of 0.115±0.001 s^−1^. A faster, minor component accounts for less than 5% of the signal change.

### Implicit Ligand Sampling (ILS)

One of the most intriguing characteristics of Ngbs is the presence of a huge cavity of several hundred Å^3^ [52]. Since the existence of this cavity involves a very high energy cost, efforts were devoted to understanding its role. Ligand migration and docking sites have been described in human Ngb by both experimental and theoretical approaches [40], [53]–[56]. To study the tunnels and docking sites of *C. aceratus* and *D. mawsoni* Ngbs, MDS and ligand (CO) migration within the protein matrix by ILS were performed as described [38]. Detailed analyses of dynamic properties of these proteins have been described elsewhere [23], [57] and we just refer to a few key findings. As expected, both Antarctic Ngbs show a huge inner cavity connected to the solvent through a few distinct pathways. Notably, in addition to the tunnel passing through the distal site and exiting by the CD corner, there are several other channels connecting docking sites to the solvent, characterised by similar and relatively low energetic barriers, as reported in human Ngb [56]. The overall shape of the cavities appears similar in the five-coordinated deoxy form. In the six-coordinated bis-histidyl and the CO conformations of Ngb, the details of the energetic profiles retrieved by ILS, are different, suggesting relevant dynamic processes affecting ligand migration and reactivity of Ngbs.

**Figure 2:**
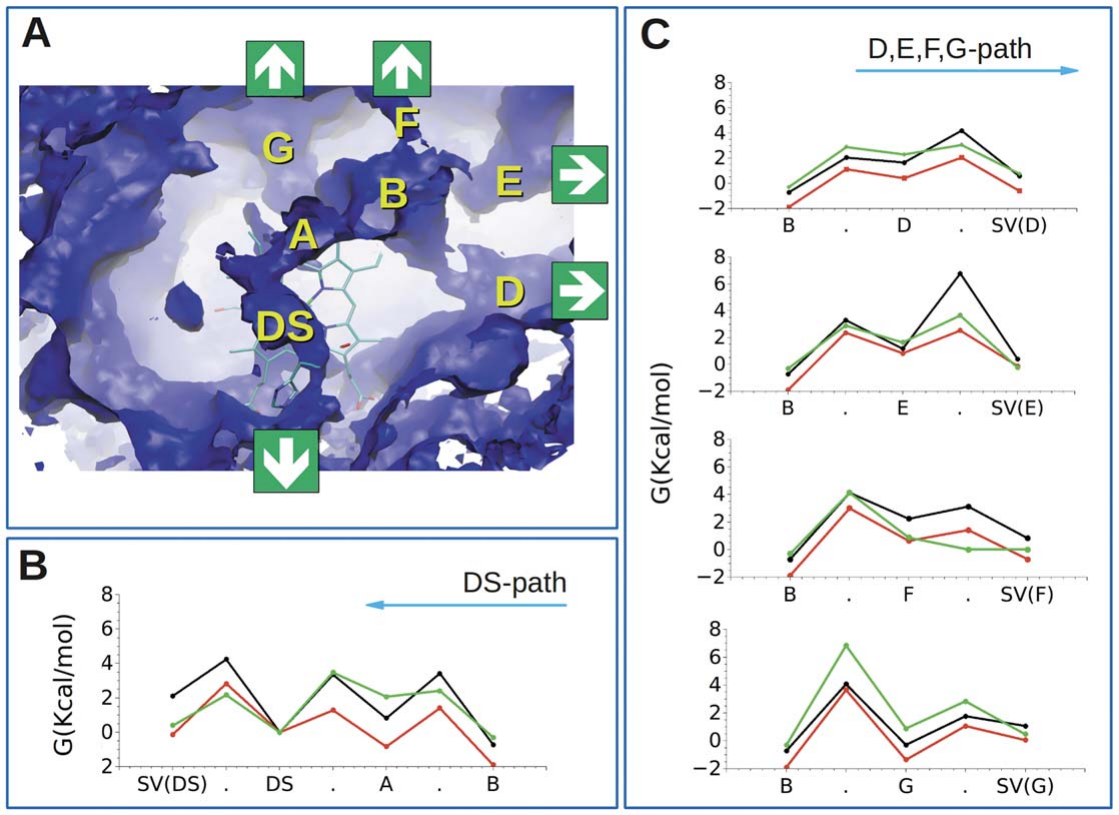
Cavities in Antarctic Ngbs. **A.** Iso surface representation of five-coordinated *C. aceratus* Ngb highlighting the distal site (DS), and A, B, D, E, F and G cavities. The heme is in sticks and putative ligand exit sites are indicated. **B.** Free**-**energy diagram of migration pathway found in five-coordinated *C. aceratus* Ngb (black), *D. mawsoni* Ngb (red) and human (green), connecting cavity B to the solvent (SV) through DS. From right to left are the energies of a ligand exiting to the solvent through the HisE7 gate, visiting the DS. **C.** Free**-**energy diagram of the alternative most favourable migration pathways connecting cavity B (left) to the solvent (SV, right). Energy barriers of a ligand exiting from B to the solvent through cavities D (top), E (upper middle), F (lower middle) and G (bottom).

The mutual position of cavities is highlighted in Figure 2A for the five-coordinated deoxy conformation of *C. aceratus* Ngb. The cavity on top of the heme (DS in Figure 2A) is connected to the solvent by a tunnel passing by the CD corner. Exit from DS through this pathway, which resembles the distal His gate in Mb, occurs *via* an energy barrier of ∼4 kcal/mol in *C. aceratus* Ngb and ∼3 kcal/mol in *D. mawsoni* Ngb (Figure 2B). These barriers are higher than in human Ngb, for which we estimate nearly 2 kcal/mol (Figure 2B). These differences may be explained by the fact that Antarctic fish Ngbs are shorter by one residue in the CD region than human Ngb [23]. This structural difference is relevant in a region such as the CD loop. The latter is significant for protein function because it affects the position and the dynamics of distal His, thus shaping the connection between the solvent and DS. As a consequence of the shorter CD loop, the Cys-Cys distance between the two Cys, that form the disulfide bridge in human Ngb, is several Å shorter than in human Ngb [23]. Thus, the protein adopts a conformation, which is in principle more suitable to form a disulfide bridge than in human Ngb, where a more important rearrangement is needed.

Cavity DS is also connected through the small cavity A to the huge cavity B, from which several tunnels depart (Figure 2A). While the barrier that ligands encounter on the path from DS to A is similar in human and *C. aceratus* Ngb, the next steps are characterised by shallower dips in human Ngb (Figure 2B). In *D. mawsoni* Ngb, DS and cavity A are separated by a lower barrier than in human Ngb and *C. aceratus* Ngb, whereas the next steps show activation energies comparable to the ones for *C. aceratus* Ngb (Figure 2B).

There are alternative pathways from cavity B to the solvent. The main ones are: (*i*) the tunnel between helices G and H, close to the GH loop (main cavity F in Figure 2A); (*ii*) the tunnel between helices G and H, close to the C terminus (main cavity G in Figure 2A); (*iii*) the tunnel between helices E and F, between the heme and the EF loop with the identified docking site D; (*iv*) the tunnel between helix A and the GH loop (main cavity E in Figure 2A). All these cavities are energetically accessible at room temperature with a probability comparable to that for the DS exit. The correspondence between ligand migration pathways studied in this work and those identified in similar investigations [56], [58] is reported in the Supporting Information (Table S2). The first barriers from B to the different cavities are equivalent in the three proteins, except for a high barrier of almost 7 kcal/mol from B to G in human Ngb (Figure 2C). The second barriers from cavities to the solvent are slightly lower in human Ngb, especially for the path through the F cavity, in contrast with slightly higher barriers in *C. aceratus* Ngb. In particular, there is a barrier of 6 kcal/mol from E to the solvent, expected to inhibit exit to the solvent through this route in *C. aceratus* Ngb (Figure 2C). The different barriers between the distal cavity and the solvent along the migration pathways mean that ligands can exploit some of the pathways to migrate from the distal pocket to the solvent, or *vice versa*. In contrast, some of the pathways inhibit such exchange.

In the six-coordinated and CO bound conformations, the DS path is blocked by the sixth ligand. Alternative ligand-entry paths from the solvent to B exist, with barriers below 5 kcal/mol (Figure S6). Notably, the corresponding barriers in the CO-coordinated species of *C. aceratus* are much lower than in the other two CO-coordinated species (Figure S6). In contrast to human Ngb, the B cavity in the six-coordinated conformation of *C. aceratus* and *D. mawsoni* Ngbs remain available and connected to the solvent through the G cavity with relatively low-energy barriers (less than 4 kcal mol^−1^) [56] (Figure S6, panel B).

The usual assumption is that ligands enter the protein in the reactive five-coordinated state. Here we show that both in the CO- and six-coordinated species, the ligand may enter with a relatively low barrier to the internal cavities. Thus, these results suggest that proteins may load ligands before His dissociation occurs and enhance protein reactivity. Alternatively, a second ligand may enter the protein in a ligand-bound state favouring multi-substrate reactions such as NO dioxygenase [6].

### Laser-flash photolysis

**Figure 3:**
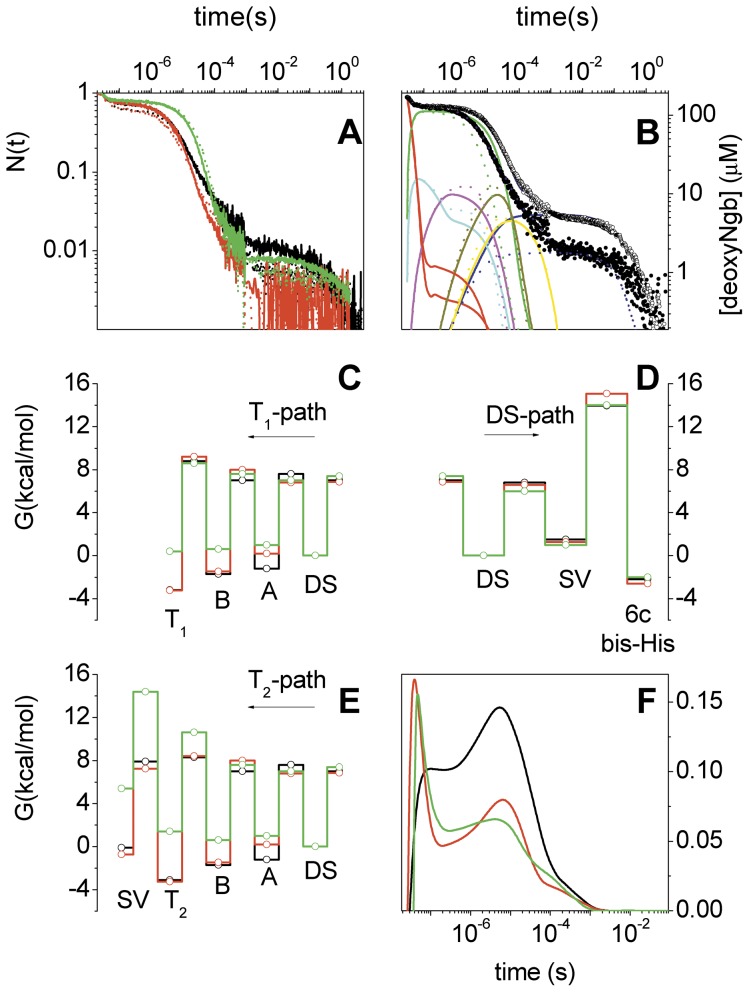
Laser-flash photolysis of *D. maw*Ngb*, *C. ace*Ngb* and human Ngb. **A.** Comparison between CO-rebinding kinetics measured at 436 nm for *D. maw*Ngb* (red), *C. ace*Ngb* (black) and human Ngb (green) at 20°C (solid lines) and 10°C (dotted lines). Solutions were equilibrated with 1 atm CO. **B.** Fitting of CO-rebinding curves to *C. ace*Ngb* T = 20°C, 1 atm (filled circles), 0.1 atm (open circles). Reaction intermediates are also reported as solid and dotted lines for data taken at 1 atm CO and 0.1 atm CO, respectively. **C, D, E.** Free-energy profiles at 20°C for ligand migration through the internal cavities, ligand exit to the solvent from the distal pocket, and six-coordination by distal His. In black, *C. ace*Ngb*; in red, *D. maw*Ngb*; in green, human Ngb. **F.** Time course of the fraction of photodissociated ligands migrating through cavities as estimated from the fitting with Scheme 1. In black, *C. ace*Ngb*; in red, *D. maw*Ngb*; in green, human Ngb. T = 20°C, 1 atm CO.

CO rebinding after laser photolysis of Ngbs occurred with multiphase kinetics, in which specific contributions can be identified, on the basis of previous experiments on human and murine Ngbs [59], [60]. Figure 3A compares the CO-rebinding kinetics measured in *C. ace*Ngb*, *D. maw*Ngb*, and human wt Ngb, following nanosecond laser photolysis. Experiments at different CO concentrations, e.g. the one reported in Figure 3B for *C. ace*Ngb*, allowed to distinguish between the geminate and the second-order rebinding phases. When liganded Ngb is photodissociated, there is a period of time during which the photodissociated ligands remain within the protein. This composite species is referred to as the ‘geminate pair’. The geminate pair can decay either through escape of the ligand into the bulk solvent or by recombining with the heme iron to which it was originally bound. The geminate phase in ligand-rebinding kinetics to Ngb exposed a series of ligation intermediates, consistent with the presence of a discrete series of temporary docking sites (see above), through which the photodissociated ligand migrates. The results indicated that geminate recombination in *C. ace*Ngb* and *D. maw*Ngb* was similar and remarkably larger than the corresponding phase in human Ngb, probably as a result of a hindered escape route for photodissociated ligands. The extent of the six-coordinated species, formed after photodissociation in a competitive reaction with CO rebinding, was different in the three proteins. This is clearly seen from the data at 20°C in Figure 3A, in which the six-coordinated species was produced in higher yield for *C. ace*Ngb*. As judged from the residual absorbance at ≈10 ms, heme hexacoordination by distal HisE7 competes with CO rebinding with the highest efficiency. While rebinding was extended in time in all samples, we observed a broader second-order phase in *D. maw*Ngb* and to a larger extent in *C. ace*Ngb* than in human Ngb. Broadening was mostly evident in an additional decay at about 200 µs, whose rate is weakly temperature dependent, and almost CO-concentration independent. Geminate rebinding had an appreciable temperature dependence, generally proving involvement of protein dynamics in the exit of the photodissociated ligand from the distal pocket. By contrast, second-order rebinding had an apparently weaker temperature sensitivity than in human Ngb.

The above results from ILS as well as recent literature data [58] suggest that the kinetic model we have recently proposed for human Ngb based on static crystal structures [40] requires some modifications to take into account the dynamic connectivity of internal cavities. The distal cavity (DS in Figure 3A) in human Ngb and in both fish Ngbs is connected to a tunnel hosting a series of docking sites (A and B in Figure 3A). This tunnel branches into distinct pathways, labelled as D, E, F, and G in Figure 3A. The ILS results suggest that some of the tunnels may represent additional entry/exit points for ligands, through direct connections to the solvent. In contrast, ligand exchange through some of the branches appears more difficult. Thus, we adopted a branched reaction scheme (Scheme 1). As for human Ngb, the model assumes that the photodissociated ligand can either escape to the solvent (*Ngb_p_*) through a direct channel connecting the distal pocket with the solvent (the His gate), or migrate through a series of temporary docking sites. From the primary docking site in the distal pocket (DS in Figure 2B), the photodissociated ligand can sequentially access two additional binding sites (A and B in Figure 2B). Then the ligand can migrate to one of several pathways with main docking sites D, E, F, and G. All these paths are more or less connected to the solvent, through barriers of different heights. Since modelling explicitly all four migration pathways would result in heavy over-parameterisation of the kinetics, we have simplified the reaction scheme by including only two reaction branches. One is representative of those paths, characterised by a high enough barrier between the docking site *T_1_* and the solvent, to prevent exit of the ligand to an appreciable extent. A second reaction route has an explicit connection between trap *T_2_* and the solvent, through a barrier allowing some ligands to escape the protein matrix. Finally, the deoxy five-coordinated species (*Ngb_p_*) is in equilibrium with the deoxy six-coordinated bis-His species (*Ngb_h_*).
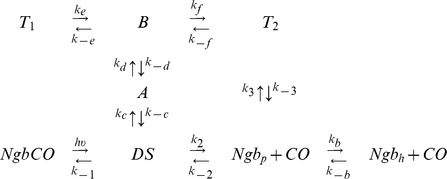

**Scheme 1.** Minimal reaction scheme for the observed kinetics with sequential migration between internal hydrophobic cavities. To allow easier comparison, we have labelled the reaction intermediates using the same symbols we used to indicate the cavities identified by ILS.

Analysis of the CO rebinding kinetics to *C. ace*Ngb*, *D. maw*Ngb* and human Ngb with the model proposed in Scheme 1 proved satisfactory in the temperature and CO-concentration ranges employed. An example of fitting under selected conditions is reported in Figure 3B for *C. ace*Ngb*, along with the time course of the reaction intermediates. Rate constants at 20°C retrieved from the fitting procedure are reported in Table 2, along with the activation free energies estimated from linear Eyring plots of the microscopic rate constants. For comparison, Table 2 also reports the corresponding values in human Ngb. While the progress curve appears equivalent to the one we have previously proposed for human Ngb [40], the current determination considers an additional piece of information from MD simulations, which at the time was not available.

**Table 2:** Microscopic rate constants from the fit of the flash photolysis data, at 20°C.

		*C. ace*Ngb*		*D. maw*Ngb*		*human*
	*k*	*ΔG* ^‡^ (kcal/mol)	*k*	*ΔG* ^‡^ (kcal/mol)	*k*	*ΔG* ^‡^ (kcal/mol)
*k*_−1_ (10^7^ s^−1^)	2.0	7.0±0.7	3.0	6.9±0.1	1.7	7.4±0.4
*k*_2_ (10^7^ s^−1^)	5.6	6.8±0.2	8.5	6.6±0.7	10.5	6.0±0.4
*k*_−2_ (10^8^ M^−1^ s^−1^)	8.3	5.4±0.9	6.6	5.3±0.9	5.5	5±1
*k*_3_ (10^3^ s^−1^)	15.9	11±3	42.0	11±9	0.85	13±5
*k*_−3_ (10^6^ M^−1^ s^−1^)	4.4	8±5	0.81	8±1	2.9	9±3
*k*_b_ (s^−1^)	2900	12±1	670	13±3	545	13±2
*k*_−b_ (s^−1^)	6.0	16±6	2.6	16±3	0.42	16±4
*k*_c_ (10^7^ s^−1^)	0.88	7.6±0.7	4.0	7±2	5.4	7±1
*k*_−c_ (10^7^ s^−1^)	0.1	8.8±0.9	4.3	6.7±0.8	12.8	6±0.8
*k*_d_ (10^6^ s^−1^)	3.0	8.2±0.8	6.0	8±1	70.2	6.6±0.9
*k*_−d_ (10^6^ s^−1^)	1.4	8.7±0.1	0.29	9.5±0.1	34.9	7.0±0.1
*k*_e_ (10^6^ s^−1^)	0.042	10.5±0.9	0.036	11±2	12	8±1
*k*_−e_ (10^5^ s^−1^)	0.026	12±6	0.031	12±5	5.5	9±5
*k*_f_ (10^5^ s^−1^)	1.4	10±4	1.4	9.9±0.5	0.85	10±2
*k*_−f_ (10^3^ s^−1^)	17.0	11.4±10	20.5	12±17	3.3	12±5

Activation free energies at 20°C were estimated from the linear Eyring plots for each rate constant *k*_i_ in the temperature range 5–20°C.

Numerical analysis demonstrated that, in *C. ace*Ngb* and *D. maw*Ngb*, the photodissociated ligand escapes to the solvent (rate *k*_2_) with lower probability than in human Ngb. Reactivity of the ligand at the primary docking site (rate *k*_−1_) is on the other hand similar to that of human Ngb. The values of these two rates account for the observed larger geminate recombination observed in *C. ace*Ngb* and *D. maw*Ngb*.

Formation of the six-coordinated species (rate *k*_b_) occurs with higher rate in *C. ace*Ngb* and leads to more efficient formation of this reaction intermediate. Dissociation of distal His from the heme also occurs with higher rate, resulting in not too dissimilar equilibrium constants. The equilibrium constants *K*_H_, determined at 20°C from the ratio of the binding (*k*_b_) and dissociation (*k*_−b_) rates (Table 2), yield 483 for *C. ace*Ngb*, 258 for *D. maw*Ngb*, and 1300 for human Ngb. The values of these equilibrium constants result in full bis-histidyl hexacoordination in the deoxy Fe^2+^ proteins.

The extent of ligand migration can be easily appreciated comparing the total relative concentrations of ligands inside inner cavities, reported in Figure 3F. Migration to the first inner cavity A is most effective in *D. maw*Ngb* and human Ngb, with a peak population at about 40 ns, although this appears to result from a different combination of microscopic rate constants (Table 2). Ligands in cavity A survive for just a few hundred nanoseconds, then quickly move on to B and the subsequent docking sites. The similarity between the time profiles reported in Figure 3F for ligand migration in *D. maw*Ngb* and human Ngb extends to about 3 µs. In contrast, migration inside *C. ace*Ngb* cavities leads to a slower, but eventually more efficient, accumulation of reactants inside the protein matrix, with a peak concentration at ∼10 µs. Interestingly, a peak is observed at this time also in the time profile for *D. maw*Ngb*. Given the combination of rates, once entering the cavity system, the ligand appears to persist within it for a slightly longer time in *C. ace*Ngb* and *D. maw*Ngb* (∼1 ms) than in human Ngb, a fact that suggests a higher stability for these locations inside fish Ngbs. Thus, from a qualitative point of view, migration through *D. maw*Ngb* cavities is somehow intermediate between human and *C. ace*Ngb*, sharing similarities with both of them.

In human Ngb, the free-energy barrier for binding to the heme, *ΔG* ^‡^ (*k*_−1_), from the primary docking site (DS) was higher and the barrier for exit to the solvent, *ΔG* ^‡^ (*k*_2_), was lower than the corresponding barriers in *C. ace*Ngb*and *D. maw*Ngb* (Figure 3, panels C, D, and E).

Further insight comes from a comparison between the free-energy barriers for migration, displayed in panels C–E of Figure 3. The barrier *ΔG* ^‡^ (*k*_c_) is similar in human Ngb and *D. maw*Ngb* and it is higher in *C. ace*Ngb*. Notably, barrier *ΔG* ^‡^ (*k*_−c_) is smaller in human Ngb than in *C. ace*Ngb*, and the corresponding barrier in *D. maw*Ngb* is intermediate between the two. The activation free energies retrieved for the remaining rate constants describing ligand migration (*k*_d_ through *k*_−f_) are very similar in *C. ace*Ngb*and *D. maw*Ngb*, both being rather different from the corresponding barriers determined in human Ngb. Thus, on short time scale after photolysis, the energetic barriers for ligand migration are similar in human Ngb and *D. maw*Ngb*, while after a few hundred nanoseconds, the barriers encountered by the diffusing ligand are more similar in *C. ace*Ngb*and *D. maw*Ngb*. Again, we point out that the behaviour of *D. maw*Ngb* shares features with both *C. ace*Ngb* and human Ngb.

A common feature to all investigated Ngbs is that the barriers increase as the ligand proceeds towards more internal cavities, a fact which may reflect coupling with concomitant conformational transitions, which ultimately result in six-coordination of the heme by distal His.

The competing reaction of the ferrous heme with the endogenous ligand, leading to bis-histidyl six-coordination, has functional consequences on the binding rate constants for exogenous ligands [61]. The binding rate constant to the ferrous five-coordinated form (*k_ON_*) is quite similar in *C. ace*Ngb* and *D. maw*Ngb*, with values of 2.2×10^8^ M^−1^ s^−1^ and 1.7×10^8^ M^−1^ s^−1^, respectively. These figures are at least twofold higher than the value of 7.7×10^7^ M^−1^ s^−1^ observed in human Ngb, a difference arising from similar rebinding (*k*_−1_) and lower escape (*k*_2_) and return (*k-*_2_) rates. *k_ON_* we estimated in human Ngb compares well to previous determinations (5.0×10^7^ M^−1^ s^−1^) [62]. Interestingly, CO binding to zebrafish Ngb occurs with *k_ON_* = 7×10^7^ M^−1^ s^−1^ [62], a value which is almost identical to the one we determined in human Ngb. CO rebinding in Antarctic fish Ngbs occurs significantly faster than in human Ngb. Since rebinding in the latter Ngb occurs at a rate which is similar to that of *Danio rerio* Ngb (see the two *k_ON_* values), the difference between human and Antarctic fish Ngbs appears relevant, and not due to the different phylogeny of the species. Further, it should be considered that binding to equilibrium in deoxy Fe^2+^ human Ngb, *C. ace*Ngb* and *D. maw*Ngb* occurs to fully bis-histidyl, six-coordinated proteins with *K*_H_ = 476 in *C. ace*Ngb*, *K*_H_ = 255 in *D. maw*Ngb*, and *K*_H_ = 1300 in human Ngb. Thus, observed binding rates to six-coordinated proteins (*k_ON,obs_*) are much lower and can be estimated from [61]:
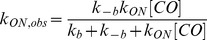
It can therefore be estimated that at 1 mM CO, the value of *k_ON,obs_* are 6 s^−1^ in *C. ace*Ngb*, 3 s^−1^ in *D. maw*Ngb*, and 0.2 s^−1^ in human Ngb. Thus, even in the presence of relatively high concentrations of gaseous ligands, these globins are expected to react rather slowly. As already pointed out for other parameters, *k_ON,obs_* of *D. maw*Ngb* is intermediate between those of *C. ace*Ngb*, which shows the highest binding rate, and human Ngb, characterised by the lowest binding rate.

## Concluding Remarks

Unlike Antarctic fish Hbs, which display different functional properties compared to HbA, all experimental and theoretical data presented herein suggest that the structural properties of Ngb, are maintained in the two Antarctic fish and between them and human Ngb.

Larger geminate recombination and faster CO rebinding in both Antarctic Ngbs compared to human Ngb suggest they may be responsible for optimisation of biological function. In contrast to red-blooded-fish Ngb, the icefish protein shows slower migration into and within the cavities, accompanied by a more efficient accumulation of ligands within the protein matrix. Migration across the cavities of *D. maw*Ngb* displays a behaviour that falls between human and *C. ace*Ngb*. While the rapid autoxidation of the oxygen-bound species suggests that Ngb has not evolved to store and supply oxygen, the presence of multiple binding sites allowing temporary docking of small gaseous ligands for relatively long times may be consistent with involvement in the NO-dependent processes, as proposed for human and mouse Ngbs [6].

Antarctic fish live at a constant temperature of −1.9°C. The high-oxygen content in the Antarctic waters led to remarkable evolutionary adaptations in fishes. Antarctic icefish survive without Hb genes and many species also fail to express Mb. In these fish, globin loss is correlated with increases in cellular mitochondrial density, heart size, blood volume and capillary bed volume. It was suggested that the high NO levels occurring in the absence of both Hb and Mb have triggered some of the major cardiovascular and sub-cellular compensations mentioned above. In cellular and tissue microenvironments, dynamic NO behaviour is strongly dependent on the action of Hb and Mb, the major drivers in scavenging NO bioactivity. Therefore, icefish represent a particularly challenging case study in analysing NO metabolism, as well as in understanding the interplay of hemoproteins with NO.

Because NO regulates physiological responses that are similar to the cardiovascular adaptations of icefishes, increasing attention is being focussed on the pathways of NO production and degradation in Antarctic notothenioids. In mammals, the molecular response to limited oxygen availability include higher expression of nitric oxide synthase (NOS) such as the type I (neuronal, nNOS) that produces an increase in NO synthesis [63]. Morlà et al. (2003) reported that five icefish species express nNOS constitutively in skeletal muscle at higher levels than those found in six red-blooded notothenioids [64]. These results are consistent with a higher NO production in icefish that may participate in mantaining a reduced peripheral resistance to blood flow. Although these data are limited, they strongly suggest that NO biology is fundamentally different in icefish compared to that of red-blooded fish.

Although the influence of NO metabolism in the icefish does not apparently result in structural and biophysical differences in Ngb properties, other mechanisms, such as gene regulation and/or protein expression, may govern Ngb adaptations in Antarctic fish under specific physiological requirements. These mechanisms have been already pointed out in other species [65]–[68] but are poorly understood in Antarctic fish. Future investigations on the role of mRNA expression in the brain and retina will identify putative differences between proteins adapted in different environments (Giordano, personal communication).

Since the icefish *C. aceratus* lacks Hb and cardiac Mb, this study was performed to investigate the differences between its Ngb and the human protein. Moreover, the comparison with a phylogenetically related red-blooded phenotype in which globin genes are functional may be a valuable system for understanding the interplay of globins in tissues.

## Supporting Information

Figure S1 **UV-visible and RR spectra of** ***D. maw*** Ngb*. UV-visible (left) and RR (right) spectra of Fe^3+^, Fe^2+^, oxy, and CO complex of *D. maw*Ngb*, in 20 mM Tris-HCl pH 7.6. The asterisks in the spectrum of the CO adduct indicate impurities. Experimental conditions are identical to those of *C. ace*Ngb* (see Figure 1).
(DOC)

Figure S2 **RR spectra in the low-frequency region of** ***D. maw*** Ngb*. RR spectra in the low- frequency region of Fe^2+^, and CO complex of *D. maw*Ngb*, in 20 mM Tris-HCl pH 7.6. Experimental conditions are identical to those of *C. ace*Ngb* (see Figure 1).
(DOC)

Figure S3 **Autoxidation of the oxygenated forms of** ***C. ace*** Ngb* (green circles), ***D. maw*** Ngb*(red circles) and human Ngb (black circles). The reaction was monitored at 580 nm and the traces were normalised using a spectrum collected at 4°C immediately after exposure to oxygen and a spectrum obtained in the presence of sodium ferricyanide as references for the pure oxy- and met- forms, respectively. Red solid lines are the result of the best fit to single exponential decay functions.
(DOC)

Figure S4 **RR spectra in the high-frequency region of the Fe^2+^ form, its CO complex, and the photolysed-CO product of** ***C. ace*** Ngb*. Experimental conditions for the Fe^2+^ and CO-adduct (20 mM Tris-HCl pH 7.6) are as reported in Figure 1. Photolysed-CO: 25 mW laser power at the sample, average of 2 spectra with 240-sec integration time (high- and low-frequency regions). Spectra have been shifted along the ordinate axis to allow better visualisation. The low-frequency region has been expanded 2.5-fold.
(DOC)

Figure S5 **CO dissociation kinetics of human Ngb,** ***C. ace*** Ngb* and ***D. maw*** Ngb*. The Fe^2+^ complexes with CO were reacted treated with excess NO.
(DOC)

Figure S6 **Energy profile of migration pathways connecting cavity B to the solvent (SV) in CO-coordinated (A) and six-coordinated (B) species.** From left to right: energy barriers of a ligand exiting from B to the solvent through cavities D (top), E (upper middle), F (lower middle) and G (bottom). In black, *C. ace*Ngb*; in red, *D. maw*Ngb*; in green, human Ngb.
(DOC)

Table S1 **Normal mode assignments of the RR band of** ***C. ace*** Ngb* and ***D. maw*** Ngb*. Normal mode assignments of the RR band (in cm^−1^) observed in the high-wavenumber region of the Fe^3+^, Fe^2+^ forms together with the oxy and CO adducts of *C. ace*Ngb* and *D. maw*Ngb*.
(DOC)

Table S2 **Comparison of topologically similar ligand migration pathways in different works.** Correspondence between ligand migration pathways identified in this work and those in [56] and [58].
(DOC)

Text S1 **Cloning and sequencing of Ngb cDNA.** To obtain notothenioid-specific primers for Ngb cDNA, a partial Ngb gene was PCR-amplified from genomic DNA of several Antarctic notothenioid species with a pair of primers designed to conserved regions in teleost Ngb sequences available in the database. Sequencing of the PCR product from each species confirmed that we were dealing with Ngb genomic sequences.
(DOC)
