# Supplementary material for: Biophysical Characterisation of Neuroglobin of the Icefish, a Natural Knockout for Hemoglobin and Myoglobin. Comparison with Human Neuroglobin
Source: PLoS One. 2012 Dec 3;7(12):e44508. doi: 10.1371/journal.pone.0044508 (PMC3513292; doi:10.1371/journal.pone.0044508)
Supplement: Figure S1 — UV-visible and RR spectra of D. maw Ngb*. UV-visible (left) and RR (right) spectra of Fe3+, Fe2+, oxy, and CO complex of D. mawNgb*, in 20 mM Tris-HCl pH 7.6. The asterisks in the spectrum of the CO adduct indicate impurities. Experimental conditions are identical to those of C. aceNgb* (see Figure 1). (DOC) [file pone.0044508.s001.doc]

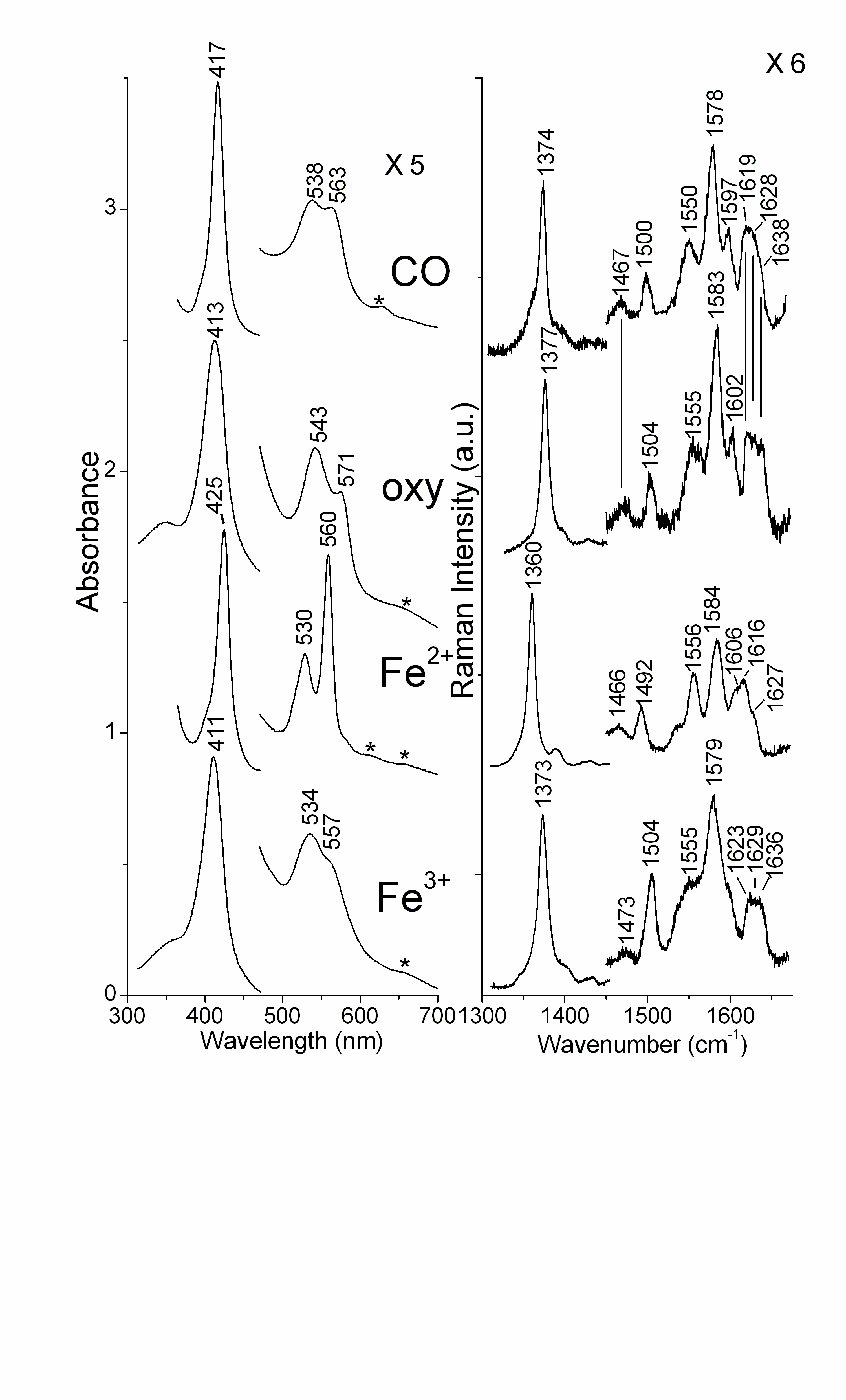


**Figure S1.** **UV-visible and RR spectra of *D. maw*Ngb*.** UV-visible (left) and RR (right) spectra of Fe^3+^, Fe^2+^, oxy, and CO complex of *D. maw*Ngb*, in 20 mM TRIS-HCl pH 7.6. The asterisks in the spectrum of the CO adduct indicate impurities. Experimental conditions are identical to those of *C. ace*Ngb* (see Figure 1).
