# Supplementary material for: Biophysical Characterisation of Neuroglobin of the Icefish, a Natural Knockout for Hemoglobin and Myoglobin. Comparison with Human Neuroglobin
Source: PLoS One. 2012 Dec 3;7(12):e44508. doi: 10.1371/journal.pone.0044508 (PMC3513292; doi:10.1371/journal.pone.0044508)
Supplement: Figure S2 — RR spectra in the low-frequency region of D. maw Ngb*. RR spectra in the low- frequency region of Fe2+, and CO complex of D. mawNgb*, in 20 mM Tris-HCl pH 7.6. Experimental conditions are identical to those of C. aceNgb* (see Figure 1). (DOC) [file pone.0044508.s002.doc]

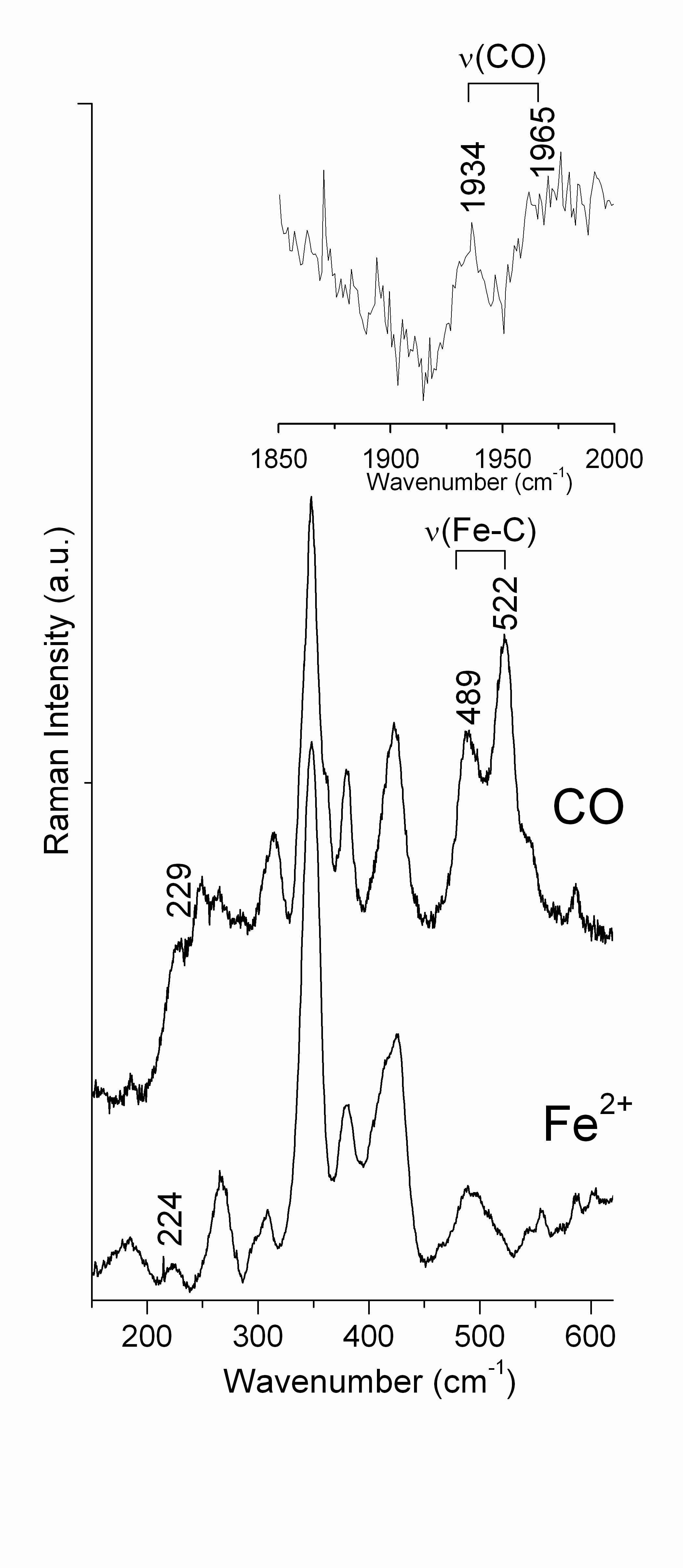


**Figure S2. RR spectra in the low-frequency region of** ***D. maw*Ngb*.** RR spectra in the low- frequency region of Fe^2+^, and CO complex of *D. maw*Ngb*, in 20 mM TRIS-HCl pH 7.6. Experimental conditions are identical to those of *C. ace*Ngb* (see Figure 1).
