# Supplementary material for: Biophysical Characterisation of Neuroglobin of the Icefish, a Natural Knockout for Hemoglobin and Myoglobin. Comparison with Human Neuroglobin
Source: PLoS One. 2012 Dec 3;7(12):e44508. doi: 10.1371/journal.pone.0044508 (PMC3513292; doi:10.1371/journal.pone.0044508)
Supplement: Figure S3 — Autoxidation of the oxygenated forms of C. ace Ngb* (green circles), D. maw Ngb*(red circles) and human Ngb (black circles). The reaction was monitored at 580 nm and the traces were normalised using a spectrum collected at 4°C immediately after exposure to oxygen and a spectrum obtained in the presence of sodium ferricyanide as references for the pure oxy- and met- forms, respectively. Red solid lines are the result of the best fit to single exponential decay functions. (DOC) [file pone.0044508.s003.doc]

**Figure S3**. **Autoxidation of the oxygenated forms of *C. ace*Ngb* (black circles), *D. maw*Ngb* (red circles) and human Ngb (green circles).** The reaction was monitored at 580 nm and the traces were normalised using a spectrum collected at 4°C immediately after exposure to oxygen and a spectrum obtained in the presence of sodium ferricyanide as references for the pure oxy- and met- forms, respectively. Black solid lines are the result of the best fit to single exponential decay functions.
