# Supplementary material for: Biophysical Characterisation of Neuroglobin of the Icefish, a Natural Knockout for Hemoglobin and Myoglobin. Comparison with Human Neuroglobin
Source: PLoS One. 2012 Dec 3;7(12):e44508. doi: 10.1371/journal.pone.0044508 (PMC3513292; doi:10.1371/journal.pone.0044508)
Supplement: Figure S4 — RR spectra in the high-frequency region of the Fe2+ form, its CO complex, and the photolysed-CO product of C. ace Ngb*. Experimental conditions for the Fe2+ and CO-adduct (20 mM Tris-HCl pH 7.6) are as reported in Figure 1. Photolysed-CO: 25 mW laser power at the sample, average of 2 spectra with 240-sec integration time (high- and low-frequency regions). Spectra have been shifted along the ordinate axis to allow better visualisation. The low-frequency region has been expanded 2.5-fold. (DOC) [file pone.0044508.s004.doc]

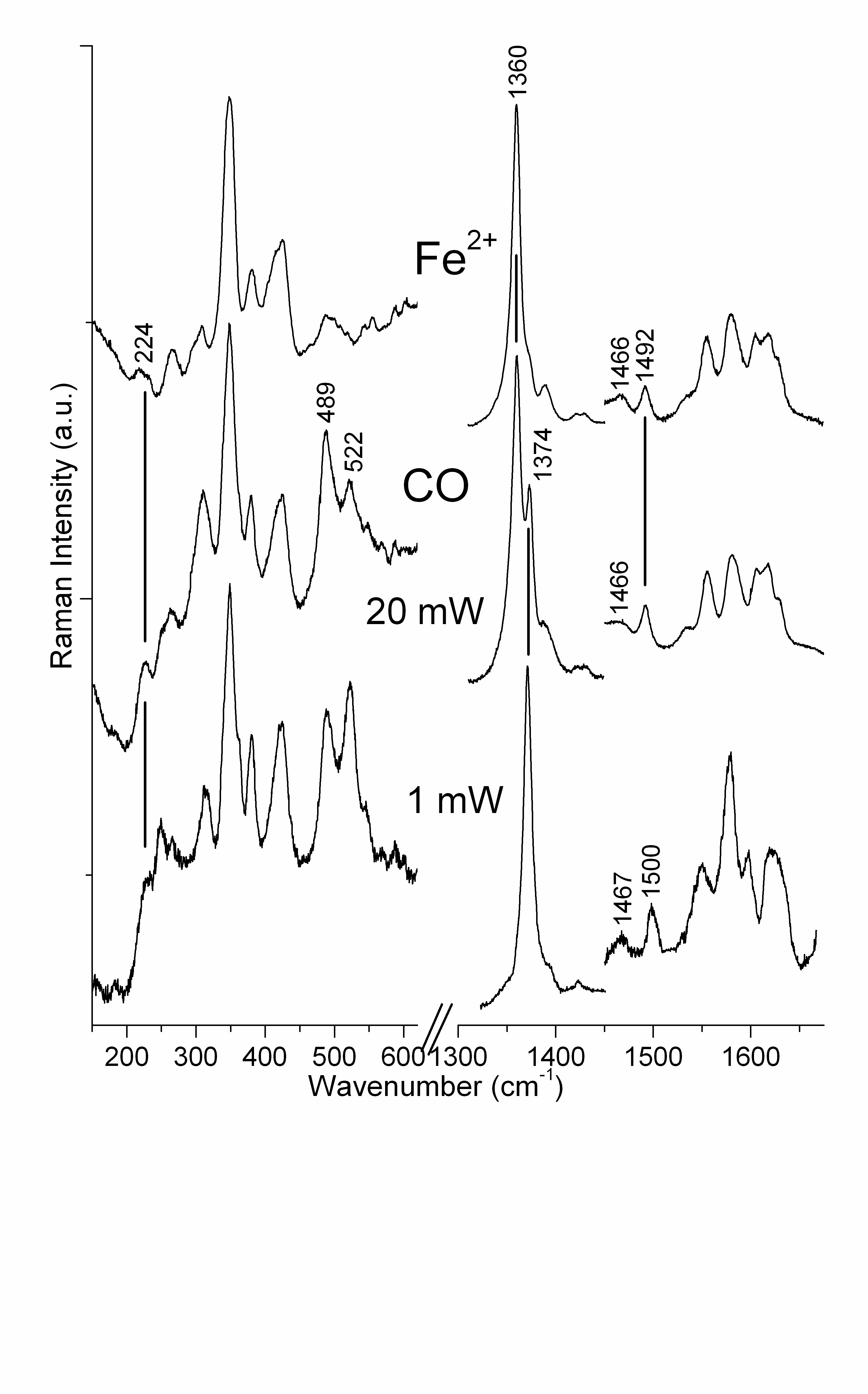


**Figure S4**. **RR spectra in the high-frequency region of the Fe^2+^ form, its CO complex, and the photolysed-CO product of *C. ace*Ngb*.** Experimental conditions for the Fe^2+^ and CO-adduct (20 mM Tris-HCl pH 7.6) are as reported in Figure 1. Photolysed-CO: 25 mW laser power at the sample, average of 2 spectra with 240-sec integration time (high- and low-frequency regions). Spectra have been shifted along the ordinate axis to allow better visualisation. The low-frequency region has been expanded 2.5-fold.
