# Supplementary material for: Biophysical Characterisation of Neuroglobin of the Icefish, a Natural Knockout for Hemoglobin and Myoglobin. Comparison with Human Neuroglobin
Source: PLoS One. 2012 Dec 3;7(12):e44508. doi: 10.1371/journal.pone.0044508 (PMC3513292; doi:10.1371/journal.pone.0044508)
Supplement: Figure S5 — CO dissociation kinetics of human Ngb, C. ace Ngb* and D. maw Ngb*. The Fe2+ complexes with CO were reacted treated with excess NO. (DOC) [file pone.0044508.s005.doc]

**Figure S5.** **CO dissociation kinetics of human Ngb, *C. ace*Ngb*and *D. maw*Ngb* .** The Fe^2+^ complexes with CO were treated with excess NO. In black, *C. ace*Ngb*; in red, *D. maw*Ngb*; in green, human Ngb.
