# Supplementary material for: Biophysical Characterisation of Neuroglobin of the Icefish, a Natural Knockout for Hemoglobin and Myoglobin. Comparison with Human Neuroglobin
Source: PLoS One. 2012 Dec 3;7(12):e44508. doi: 10.1371/journal.pone.0044508 (PMC3513292; doi:10.1371/journal.pone.0044508)
Supplement: Figure S6 — Energy profile of migration pathways connecting cavity B to the solvent (SV) in CO-coordinated (A) and six-coordinated (B) species. From left to right: energy barriers of a ligand exiting from B to the solvent through cavities D (top), E (upper middle), F (lower middle) and G (bottom). In black, C. aceNgb*; in red, D. mawNgb*; in green, human Ngb. (DOC) [file pone.0044508.s006.doc]

**
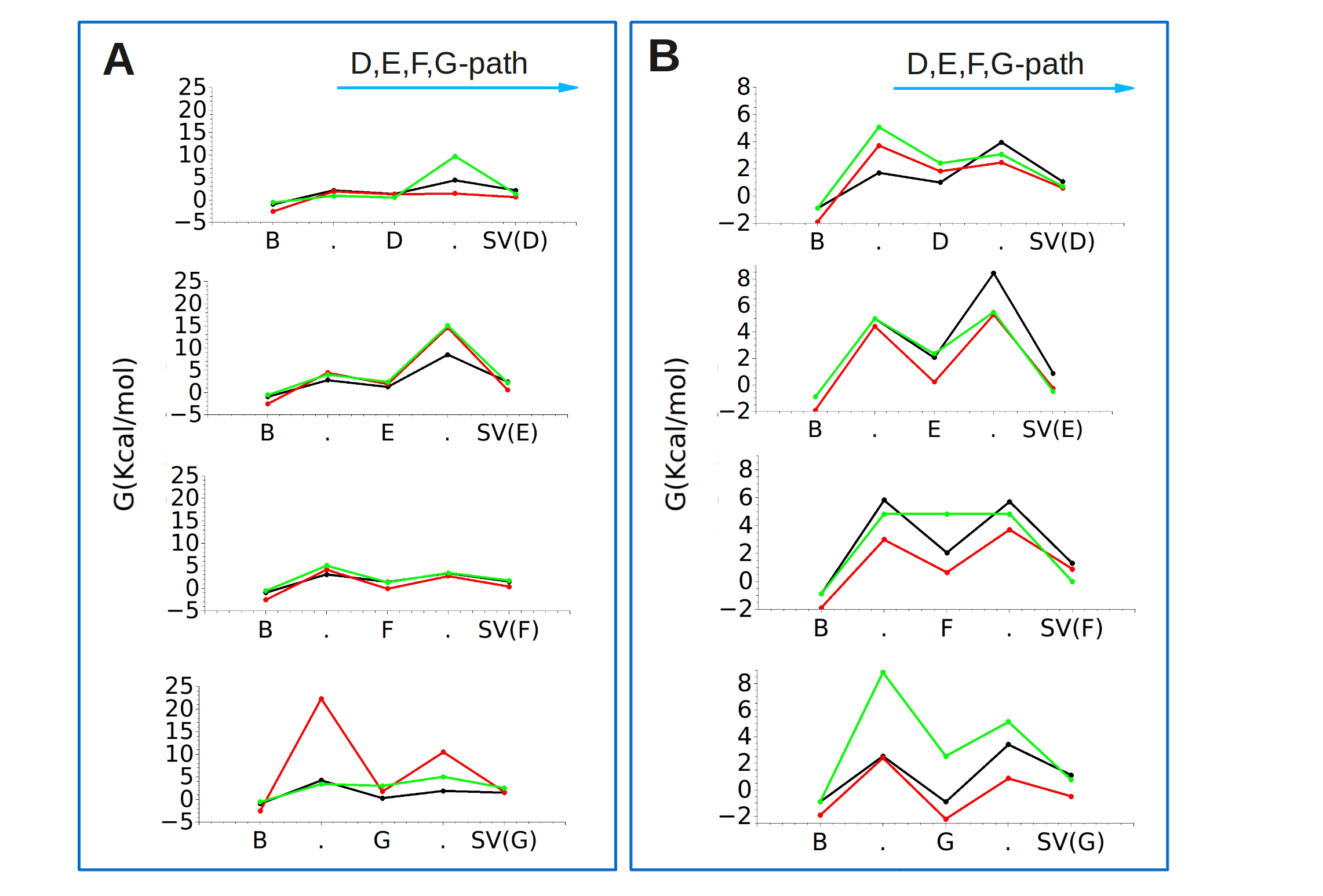
**

**Figure S6.** **Energy profile of migration pathways connecting cavity B to the solvent (SV) in CO-coordinated (A) and six-coordinated (B) species.** From left to right: energy barriers of a ligand exiting from B to the solvent through cavities D (top), E (upper middle), F (lower middle) and G (bottom). In black, *C. ace*Ngb*; in red, *D. maw*Ngb*; in green, human Ngb.
