# Supplementary material for: Biophysical Characterisation of Neuroglobin of the Icefish, a Natural Knockout for Hemoglobin and Myoglobin. Comparison with Human Neuroglobin
Source: PLoS One. 2012 Dec 3;7(12):e44508. doi: 10.1371/journal.pone.0044508 (PMC3513292; doi:10.1371/journal.pone.0044508)
Supplement: Table S1 — Normal mode assignments of the RR band of C. ace Ngb* and D. maw Ngb*. Normal mode assignments of the RR band (in cm−1) observed in the high-wavenumber region of the Fe3+, Fe2+ forms together with the oxy and CO adducts of C. aceNgb* and D. mawNgb*. (DOC) [file pone.0044508.s007.doc]

**Table S1.** **Normal mode assignments of the RR band of *C. ace*Ngb* and *D. maw*Ngb*.** Normal mode assignments of the RR band (in cm^-1^) observed in the high-wavenumber region of the Fe^3+^, Fe^2+^ forms together with the oxy and CO adducts of *C. ace*Ngb* and *D. maw*Ngb*

| Mode | Sym | **Fe^3+^** | **Fe^2+^** | **Fe^2+^-oxy** | **Fe^2+^-CO** |
| --- | --- | --- | --- | --- | --- |
| **ν_10_** | B_1g_ | 1636 | overlapped^a^ | 1638 | 1638 |
| **ν_(C=C)_** |  | 1629 | 1627 | 1628 | 1628 |
| **ν_(C=C)_** |  | 1623 | 1616 | 1619 | 1619 |
| **ν_37_** | Eu |  | 1606 | 1602 | 1597 |
| **ν_2_** | A_1g_ | 1579 | 1584 | 1583 | 1578 |
| **ν_19_** | A_2g_ |  |  | 1555 | 1550 |
| **ν_38_** | E_u_ | 1555 | 1556 |  |  |
| **ν_3_** | A_1g_ | 1504 | 1492 | 1504 | 1500 |
| **ν_28_** | B_2g_ | 1473 | 1466 | 1467 | 1467 |
| **ν_4_** | A_1g_ | 1373 | 1360 | 1377 | 1374 |

^a^Overlapped with the ν_(C=C)_ at 1616 cm^-1^

Experimental conditions: 20 mM Tris-HCl pH 7.6. Sample concentration, about 30-35 µM.
