# Supplementary material for: Biophysical Characterisation of Neuroglobin of the Icefish, a Natural Knockout for Hemoglobin and Myoglobin. Comparison with Human Neuroglobin
Source: PLoS One. 2012 Dec 3;7(12):e44508. doi: 10.1371/journal.pone.0044508 (PMC3513292; doi:10.1371/journal.pone.0044508)
Supplement: Table S2 — Comparison of topologically similar ligand migration pathways in different works. Correspondence between ligand migration pathways identified in this work and those in [56] and [58]. (DOC) [file pone.0044508.s008.doc]

**Table S2**. **Comparison of topologically similar ligand migration pathways in different works**

| Ligand migration pathways |  |  |  |  |  | Species source | *Reference* |
| --- | --- | --- | --- | --- | --- | --- | --- |
|  | DS | E | F | G | D | icefish | This work |
|  | exit 8 | exit 3 | exit 1 | exit 4 | exit 5 | human | Table 3, [56] |
|  | - | A | - | C | - | mouse | Figure S1, [58] |
