# Supplementary material for: Biophysical Characterisation of Neuroglobin of the Icefish, a Natural Knockout for Hemoglobin and Myoglobin. Comparison with Human Neuroglobin
Source: PLoS One. 2012 Dec 3;7(12):e44508. doi: 10.1371/journal.pone.0044508 (PMC3513292; doi:10.1371/journal.pone.0044508)
Supplement: Text S1 — Cloning and sequencing of Ngb cDNA. To obtain notothenioid-specific primers for Ngb cDNA, a partial Ngb gene was PCR-amplified from genomic DNA of several Antarctic notothenioid species with a pair of primers designed to conserved regions in teleost Ngb sequences available in the database. Sequencing of the PCR product from each species confirmed that we were dealing with Ngb genomic sequences. (DOC) [file pone.0044508.s009.doc]

**Supplemental InformATION**

**Biophysical characterisation of neuroglobin of the icefish,**

**a natural knockout for hemoglobin and myoglobin.**

**Comparison with human neuroglobin**

*Daniela Giordano^1^, Ignacio Boron^2,3^, Stefania Abbruzzetti^4^,Wendy Van Leuven^5^, Francesco P. Nicoletti^6^, Flavio Forti^7^, Stefano Bruno^8^, C.-H. Christina Cheng^9^, Luc Moens^5^, Guido di Prisco^1^, Alejandro D. Nadra^2,10^, Darío Estrin^2^, Giulietta Smulevich^6,11^, Sylvia Dewilde^5^, Cristiano Viappiani^4*^, Cinzia Verde^1*^*

^1^ Institute of Protein Biochemistry, CNR, Via Pietro Castellino 111, I-80131 Naples, Italy; ^2^ Departamento de Química Biológica, Facultad de Ciencias Exactas y Naturales, Universidad de Buenos Aires, Ciudad de Buenos Aires, Argentina; ^3^ Departamento de Química Inorgánica, Analítica y Química Física/INQUIMAE-CONICET, Facultad de Ciencias Exactas y Naturales, Universidad de Buenos Aires, Ciudad Universitaria, Pabellón 2, Buenos Aires, C1428EHA, Argentina; ^4^ Department of Physics, University of Parma, NEST Istituto Nanoscienze-CNR, Parma, Italy; ^5^ Department of Biomedical Sciences, PPES, University of Antwerp, Universiteitsplein 1, B-2610 Wilrijk, Belgium; ^6^ Dipartimento di Chimica “Ugo Schiff”, Università di Firenze, Via della Lastruccia 3-13, 50019, Sesto Fiorentino (FI), Italy; ^7^ Facultat de Farmacia, Departament de Fisicoquímica and Institut de Biomedicina, Universitat de Barcelona, Av. Diagonal 643, E-08028, Barcelona, Spain; ^8^ Department of Biochemistry and Molecular Biology, University of Parma, Parma, Italy; ^9^ Department of Animal Biology, University of Illinois, Urbana, IL 61801, USA; ^10^ Departamento de Fisiología, Biología Molecular y Celular, Facultad de Ciencias Exactas y Naturales, Universidad de Buenos Aires, Ciudad de Buenos Aires, Argentina; ^11^ Consorzio Interuniversitario di Ricerca in Chimica dei Metalli nei Sistemi Biologici, I-70126 Bari, Italy.

*E-mail: [c.verde@ibp.cnr.it](mailto:c.verde@ibp.cnr.it) (CVe); [cristiano.viappiani@fis.unipr.it](mailto:cristiano.viappiani@fis.unipr.it) (CVi)

**Cloning and sequencing of Ngb cDNA**

To obtain notothenioid-specific primers for Ngb cDNA, a partial Ngb gene was PCR-amplified from genomic DNA of several Antarctic notothenioid species with a pair of primers, namely Ngb F1 (5’ATGGAGAAGCTGTCRGGRAAAGACAAGG3’) and NgbR1 (5’GCCCASCCTCGGCTCATGG3’) designed to conserved regions in teleost Ngb sequences available in the database. Sequencing of the PCR product from each species confirmed that we were dealing with Ngb genomic sequences. From these partial notothenioid (noto) Ngb sequences, which are highly conserved, a reverse primer noto_Ngb5’RACE1 (5’ CTGCAGCATGTAGAGAAGGG 3’) in the Ngb coding region was designed to obtain the 5’ portion of Ngb cDNA from brain or retina RNA using the 5’RACE System for Rapid Amplification of cDNA End (Invitrogen, USA). Briefly, total RNA was extracted using Ultraspec RNA isolation reagent (Biotecx, USA). Total RNA (4 μg) was primed with a lock-dock oligodT_30_ primer, and reverse-transcribed to produce first strand cDNA using Superscript II (Invitrogen, USA). The first strand cDNA was then dC-tailed at the 3’end using terminal deoxynucleotidyl transferase (Invitrogen) and 0.2 mM dCTP. The 5’ portion of Ngb cDNA was then amplified from the dC-tailed first strand cDNA using the primer pair noto_Ngb5’RACE1 and AAP (abridged anchor primer; Invitrogen). 5’RACE was successful in generating a product from *C. aceratus* brain RNA, which was cloned into the plasmid vector pGemT_easy_  (Promega, WI), sequenced and confirmed to be partial Ngb cDNA. The *C. aceratus* partial Ngb cDNA has a 5’UTR (5’ untranslated region) of 141 bp, which was used to design two forward primers, noto_Ngb5’UTR1 (5’GTGTGCATCTCTAGCCGAGGAATCC3’) and noto_Ngb5’UTR2 (5’GGAATCCTGTCTCCAACAGTTGTGTCCC3’) to pair up with a reverse primer Ngb3’UTR1 (5’GACCYCAYTCAMAGAGCAAATGTACAGCG3’) designed to conserved 3’UTR sequences in *Gasterosteus aculeatus* and *Tetraodon nigroviridis* Ngb transcripts from the database to amplify full-length Ngb cDNA from notothenioids. The primer pair noto_Ngb5’UTR1/Ngb3’UTR1, and noto_Ngb5’UTR2/Ngb3’UTR1 successfully amplified the full-length Ngb cDNA from *D. mawsoni* retina (GenBank accession number JQ684712) and *C. aceratus* brain (GenBank accession number JQ684711) first strand cDNA respectively, which was verified by cloning into pGemT_easy_  (Promega, USA) and sequencing. All sequencing reactions were carried out using BigDye® Terminator v3.1 chemistry (ABI) and electrophoresed on ABI 3730XL capillary sequencer at the W.M. Keck Center for Comparative and Functional Genomics at the University of Illinois, Urbana-Champaign.

The Ngb cDNA of *C. aceratus* and *D. mawsoni* was subcloned in the expression vector pET3a as follows. A PCR was performed on the plasmid using the 5’ primer (GCGCATATGGAGAAGCTGTCAGAGAAAG), containing an *Nde*I restriction site that covers the initiating Met codon of the globin gene, and the 3’ primer (GCGGGATCCTCAGTCGGCCTTGTCCTCCC), containing a *Bam*HI restriction site. The amplified product was cleaned and cut with *Nde*I and *Bam*HI and subsequently ligated into the equivalently cleaved expression vector pET3a.
